# Cornuside I promoted osteogenic differentiation of bone mesenchymal stem cells through PI3K/Akt signaling pathway

**DOI:** 10.1186/s13018-021-02508-0

**Published:** 2021-06-21

**Authors:** Feng Gao, Sheng-Li Xia, Xiu-Hui Wang, Xiao-Xiao Zhou, Jun Wang

**Affiliations:** 1 Shanghai University of Medicine & Health Sciences Affiliated Zhoupu Hospital, Shanghai Pudong New District Zhoupu Hospital, Shanghai, 201318 China; 2 Department of Orthopedics, Shanghai Fifth People’s Hospital Affiliated to Fudan University, No. 801, Heqing Road, Minhang District, Shanghai, 200240 China

**Keywords:** Cornuside I, Bone mesenchymal stem cells, PI3K/Akt signaling pathway

## Abstract

**Background:**

Osteoporosis is a common disease closely associated with aging. In this study, we aimed to investigate the role of Cornuside I in promoting osteogenic differentiation of bone mesenchymal stem cells (BMSCs) and the potential mechanism.

**Methods:**

BMSCs were isolated and treated with different concentrations of Cornuside I (0, 10, 30, 60 μM). Cell proliferation was analyzed by Cell Counting Kit-8 (CCK-8) assay. RNA sequencing was performed on the isolated BMSCs with control and Cornuside I treatment. Differentially expressed genes were obtained by the R software. Alkaline phosphatase (ALP) staining and Alizarin Red Staining (ARS) were performed to assess the osteogenic capacity of the NEO. qRT-PCR and western blot were used to detect the expression of osteoblast markers.

**Results:**

Cornuside I treatment significantly improved BMSC proliferation. The optimal dose of Cornuside I was 30 μM (*P* < 0.05). Cornuside I dose dependently increased the ALP activity and calcium deposition than control group (*P* < 0.05). A total of 704 differentially expressed genes were identified between Cornuside I and normal BMSCs. Cornuside I significantly increased the PI3K and Akt expression. Moreover, the promotion effects of Cornuside I on osteogenic differentiation of BMSCs were partially blocked by PI3K/Akt inhibitor, LY294002.

**Conclusion:**

Cornuside I plays a positive role in promoting osteogenic differentiation of BMSCs, which was related with activation of PI3K/Akt signaling pathway.

## Background

Osteoporosis (OP) is a systemic metabolic bone disease caused by decreased bone density and bone mass [1, 2]. OP can easily lead to fracture [3]. The estimated number of people worldwide suffering from OP will exceed 20 million by 2030 [4, 5]. Over half of Americans 50 years and older have osteoporosis or low bone mass [6, 7]. The etiology of OP is still unknown, and the pathogenesis remains unclear [8].

Bone formation is a developmental process involving the differentiation of mesenchymal stem cells (MSCs) into osteoblasts [9]. The decreased ability of osteogenic potential of osteoblasts from MSCs is the major risk of OP [10]. Thus, promoting osteogenic differentiation is an important strategy to enhance bone mineral density and slow the development of OP [11]. So far, teriparatide is one of the most effective agents to improve the osteogenic promotion drugs in clinic [12]. Other clinical drug treatments for osteoporosis mainly include bone resorption inhibitors such as bisphosphonates and calcitonin [13]. The therapeutic effects of these two drugs are still controversial [14]. Therefore, finding a stronger ideal bone regeneration cytokine has become a clinical hot issue that needs to be solved urgently.

Corni Fructus, attributed to the liver and kidney meridians, has the effect of promoting blood circulation, invigorating vital energy, and treating pain all over the body [15–17]. Corni Fructus is one of the most frequently prescribed herbs in traditional Chinese medicine formula for treatment of osteoporosis in China [18]. Cornuside I, as a main active ingredient of Corni Fructus, is an iridoid glycoside extracted from Corni Fructus. Previous study has identified that Cornuside I could be used for treatment of OP [19]. However, the role and mechanism of Cornuside I in promoting osteogenic differentiation of BMSCs were still unknown. RNA-sequencing (RNA-Seq) technology is a high-throughput sequencing technology which has developed rapidly in recent years. RNA-Seq could be used to identify the differentially expressed genes between treatment and control groups, and thus was applied to identify the mechanism of Cornuside I in promoting osteogenic differentiation of BSMCs.

However, the mechanism through which Cornuside I promoted osteogenic differentiation of BMSCs is unclear. In this study, we firstly performed RNA sequencing to identify the mechanism of Cornuside I in promoting osteogenic differentiation of BMSCs. Then, we performed a series of studies to identify the mechanism of Cornuside I in promoting osteogenic differentiation of BMSCs.

## Material and methods

### BMSC isolation and identification

Human bone marrow mesenchymal stem cells were generated as described previously with minor modifications. In brief, the bone marrow was diluted by adding an equal volume of DMEM containing 10% fetal bovine serum (FBS). Bone marrow was then immediately centrifuged at 100×*g* for 10 min at room temperature. Then, cells were repeatedly blown and beaten the cell mass with a pipette to disperse the mass as much as possible. Cells were then cultured in a 37°C, 5% CO_2_ incubator and changed every 48 h subsequently. The identification of BMSCs was conducted by evaluating their adipogenic, osteogenic, and chondrogenic differentiation potential. In brief, BMSCs were cultured into the adipogenic, osteogenic, and chondrogenic medium (Cyagen, Guangzhou, China) to identify the tri-lineage differentiation capacity. Adipogenic, osteogenic, and chondrogenic differentiation potential were stained with Oil-Red-O staining, ARS, and Alcian blue staining respectively.

### CCK-8 assay

BMSCs (5×10^3^ cells/well) were cultured overnight, and then treated as follows: control, Cornuside I (10, 30, and 60 μM). Subsequently, CCK-8 reagents (10 μl; Beyotime, Shanghai, China) were added to BMSCs for 2 h. The absorbency of the samples was measured with a microplate reader (Bio-Rad, Richmond, CA, USA) at a wavelength of 450 nm.

### RNA sequencing

RNA was reversed transcribed into cDNA and then labeling. Chip hybridization was performed using an Affymetrix (Thermo Fisher Scientific, Inc.) expression profile chip and GeneChip Hybridization Wash and Stain kit. The results were scanned with an Agilent microarray scanner and read with the Feature Extraction software 10.7.

### Bioinformatic analysis

Differentially expressed gene (DEG) analysis was performed with the R software, using package Bioconductor package, edgeR. |logFC | >1 and P value < 0.05 were set as the threshold for screening the DEGs. The volcano plot and heatmap analysis of regulated genes were generated by using the R software, version 3.3.2. Gene ontology (GO) and Kyoto Encyclopedia of Genes and Genomes (KEGG) pathway enrichment analysis was done using DAVID bioinformatics resource portal. Gene Ontology consisted of biological process (BP), molecular function (MF), and cellular component (CC). Protein–protein interaction analysis was performed via Search Tool for the Retrieval of Interacting Genes tool (http://string-db.org/). The Cytoscape plug-in Molecular Complex Detection (MCODE, http://apps.cytoscape.org/apps/mcode) was employed to analyze modules.

### ALP and ARS

Osteogenic differentiation capacity was identified by alkaline phosphatase (ALP) staining using an ALP staining kit according to the manufacturer’s protocol. In brief, BMSCs in control and different treatment groups were fixed by 4% paraformaldehyde. Then, ALP staining solution (Millipore, UK) was added. BMSCs were then washed three times.

ARS was performed according to the manufacturer’s protocol. In brief, BMSCs were fixed by 4% paraformaldehyde and then washed with PBS for three times. Then, BMSCs were added 0.1% ARS solution (Solarbio, Beijing, China) to identify the osteogenic differentiation capacity of BMSCs. To analyze ARS activity, the ARS in stained cells was destained with 10% cetylpyridinium chloride (CPC) monohydrate solution (Sigma) for 30 min with shaking. The sections were observed under an optical microscope with a coupled digital camera (DM750, Leica, Wetzlar, DE, Germany).

### Real-time polymerase chain reaction (RT-PCR)

The TRIzol method (Invitrogen, Carlsbad, CA, USA) was adopted to extract the total cellular RNA, which was reversely transcribed into cDNA using an RT reagent kit (Takara, Japan). The reaction system was prepared with pre-denaturation at 94°C for 30 s, denaturation at 94°C for 5 s, annealing at 60°C for 15 s, and extension at 72°C for 10 s, and 45 cycles were amplified. Results were quantified using the comparative threshold method. The quantitative copy number of the target gene = 2^−ΔΔCt^. The copy number of the target gene was calculated for each specimen. All the fluorescence data were converted into relative quantification, and GAPDH was the internal contrast of RUNX2, OCN, and Osterix. The primers were designed and synthesized by Guangzhou Ribo Technology Co., Ltd.

### Western blot

The cells were washed by cold PBS 3 times; then, 150 μL RIPA lysate (Beyotime Biotechnology, Shanghai, China) was added. The cells were lysed in ice water by ultrasound, and the protein content was determined by the Bradford method. An equal amount of proteins was taken from each group for 10% SDS-PAGE, and the proteins on the gel were transferred to PVDF membranes (Millipore, Bedford, MA, USA). The membranes were blocked at 4°C for 1 h and then incubated at 4°C overnight with the following primary antibodies: anti-RUNX2 antibody (1:500; Abcam, USA), anti-Osterix antibody (1:1000; Abcam, USA), anti-OCN antibody (1:500; Abcam, USA), anti-PI3K antibody (1:1000; Abcam, USA), anti-Akt antibody (1:300; Abcam, USA), anti-p-PI3K antibody (1:500; Abcam, USA), anti-p-Akt antibody (1:100; Abcam, USA), and anti-GAPDH antibody (1:1000; Abcam, USA). After being cleaned twice with TBST, the membranes were incubated at room temperature for 1 h with fluorescein-labeled goat anti-rabbit IgG (ab205718, 1:2000). The membrane was visualized with an ECL detection kit (Millipore, Bedford, MA, USA) using a chemiluminescence imaging system (Millipore).

### Statistical analysis

Data are presented as the mean ± standard deviation. In addition, comparisons between two groups were analyzed by the unpaired Student’s t-test. One-way analysis of variance and Tukey’s post hoc tests were used for comparisons between ≥3 groups. P<0.05 was considered to indicate a statistically significant difference.

## Results

### Identification of BMSCs

**Fig. 1:**
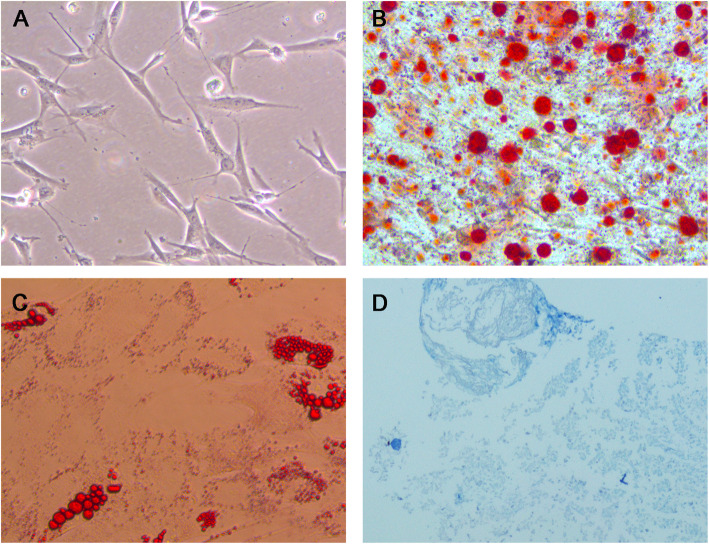
Identification of the BMSCs

BMSCs are long and spindle shaped in appearance (Fig. 1A). The BMSCs were directly induced to form osteoblasts (Fig. 1B), chondrocytes (Fig. 1C), and adipocytes (Fig. 1D) in osteogenic, chondrogenic, and adipogenic induction medium.

### Identified differentially expressed miRNAs between OA and normal samples

**Fig. 2:**
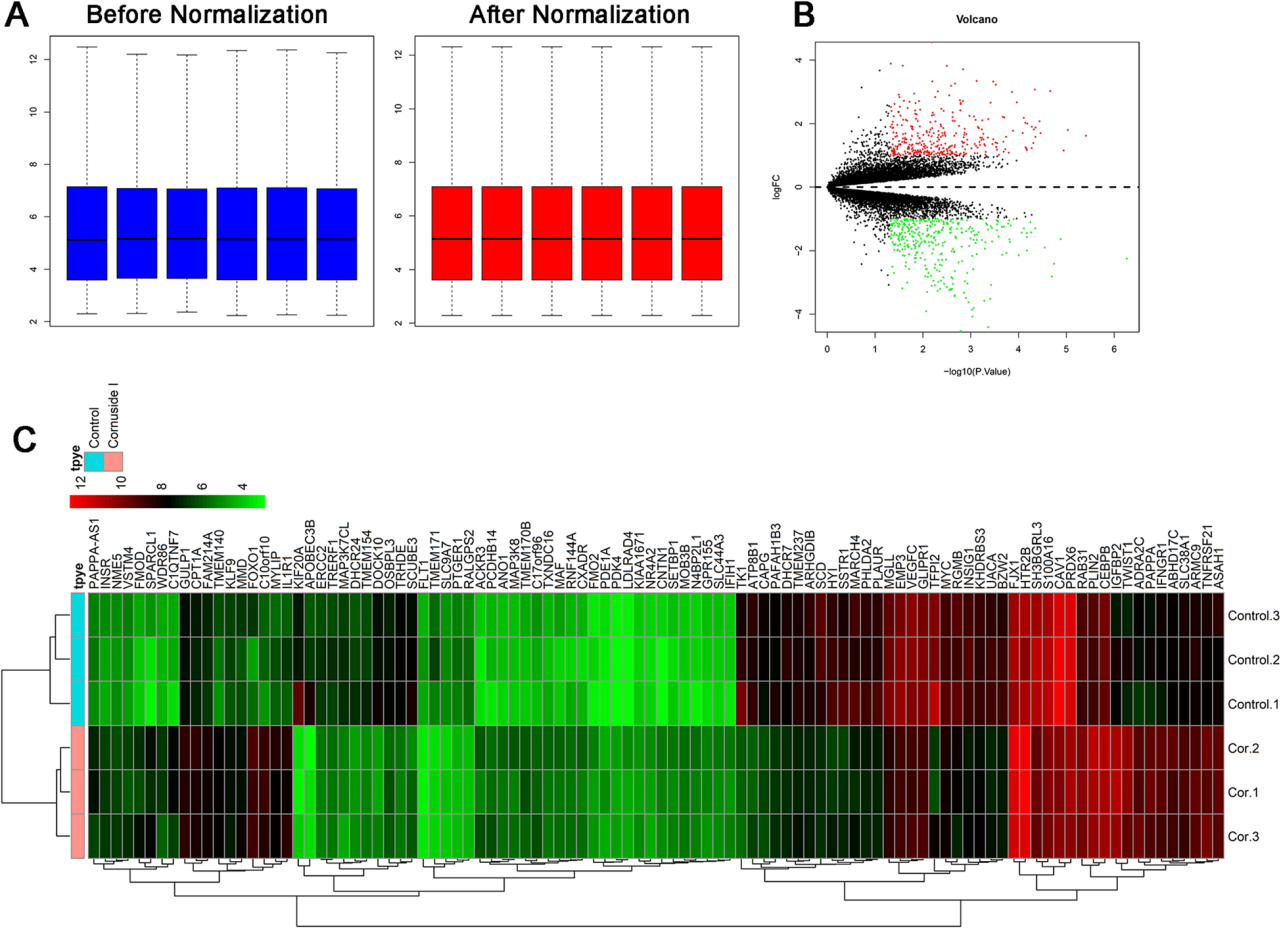
Differentially expressed genes between control and Cornuside I-treated BMSCs. **A** Expression data before normalization and after normalization. **B** Volcano plot of the differentially expressed genes between control and Cornuside I-treated BMSCs, red dots represent upregulated genes, green dots represent downregulated genes, and black dots represent non-differentially expressed genes. **C** Heatmap of the differentially expressed genes between control and Cornuside I-treated BMSCs. Red represents the upregulated genes, and green represents the downregulated genes

To analyze the differentially expressed mRNAs between normal and Cornuside I-treated BMSCs, RNA sequencing profile was subjected to bioinformatic analysis. Firstly, gene expression data were normalized using quantile normalization followed by inverse normal transformation. After normalization, the expression values were identical and could be used for further study (Fig. 2A). A total of 704 differentially expressed genes were identified between Cornuside I and normal BMSCs. Volcano plot and heatmap of the differentially expressed mRNAs can be seen in Fig. 2B and C respectively.

### GO and KEGG pathway analysis of the differentially expressed genes

**Fig. 3:**
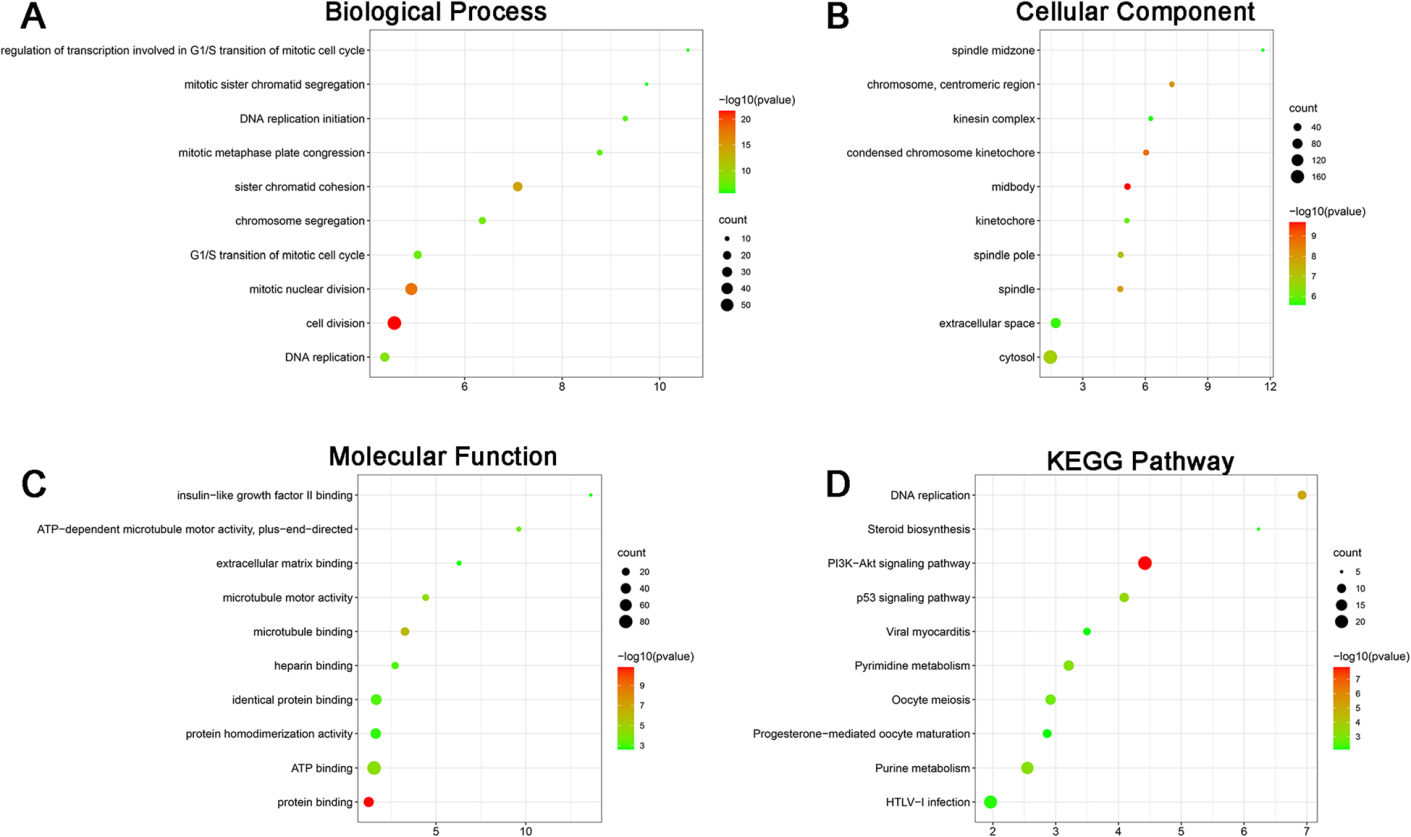
Gene ontology and KEGG pathway analysis of the differentially expressed genes. **A** Biological process of the differentially expressed genes between control and Cornuside I-treated BMSCs. **B** Cellular component of the differentially expressed genes between control and Cornuside I-treated BMSCs. **C** Molecular function of the differentially expressed genes between control and Cornuside I-treated BMSCs. **D** KEGG pathway analysis of the differentially expressed genes between control and Cornuside I-treated BMSCs

The BP of the differentially expressed genes was as follows (Fig. 3A): cell division, mitotic nuclear division, sister chromatid cohesion, DNA replication, chromosome segregation, G1/S transition of mitotic cell cycle, mitotic metaphase plate congression, DNA replication initiation, regulation of transcription involved in G1/S transition of mitotic cell cycle, and mitotic sister chromatid segregation. The MF of the differentially expressed genes was as follows (Fig. 3B): midbody, condensed chromosome kinetochore, chromosome, centromeric region, spindle, spindle pole, cytosol, kinetochore, extracellular space, kinesin complex, and spindle midzone. The CC of the differentially expressed genes was as follows (Fig. 3C): protein binding, microtubule binding, microtubule motor activity, ATP binding, ATP-dependent microtubule motor activity, plus-end-directed, identical protein binding, heparin binding, protein homodimerization activity, extracellular matrix binding, and insulin-like growth factor II binding. The KEGG pathway of the differentially expressed genes was as follows (Fig. 3D): PI3K-Akt signaling pathway, DNA replication, p53 signaling pathway, purine metabolism, pyrimidine metabolism, oocyte meiosis, HTLV-I infection, viral myocarditis, steroid biosynthesis, and progesterone-mediated oocyte maturation.

**Fig. 4:**
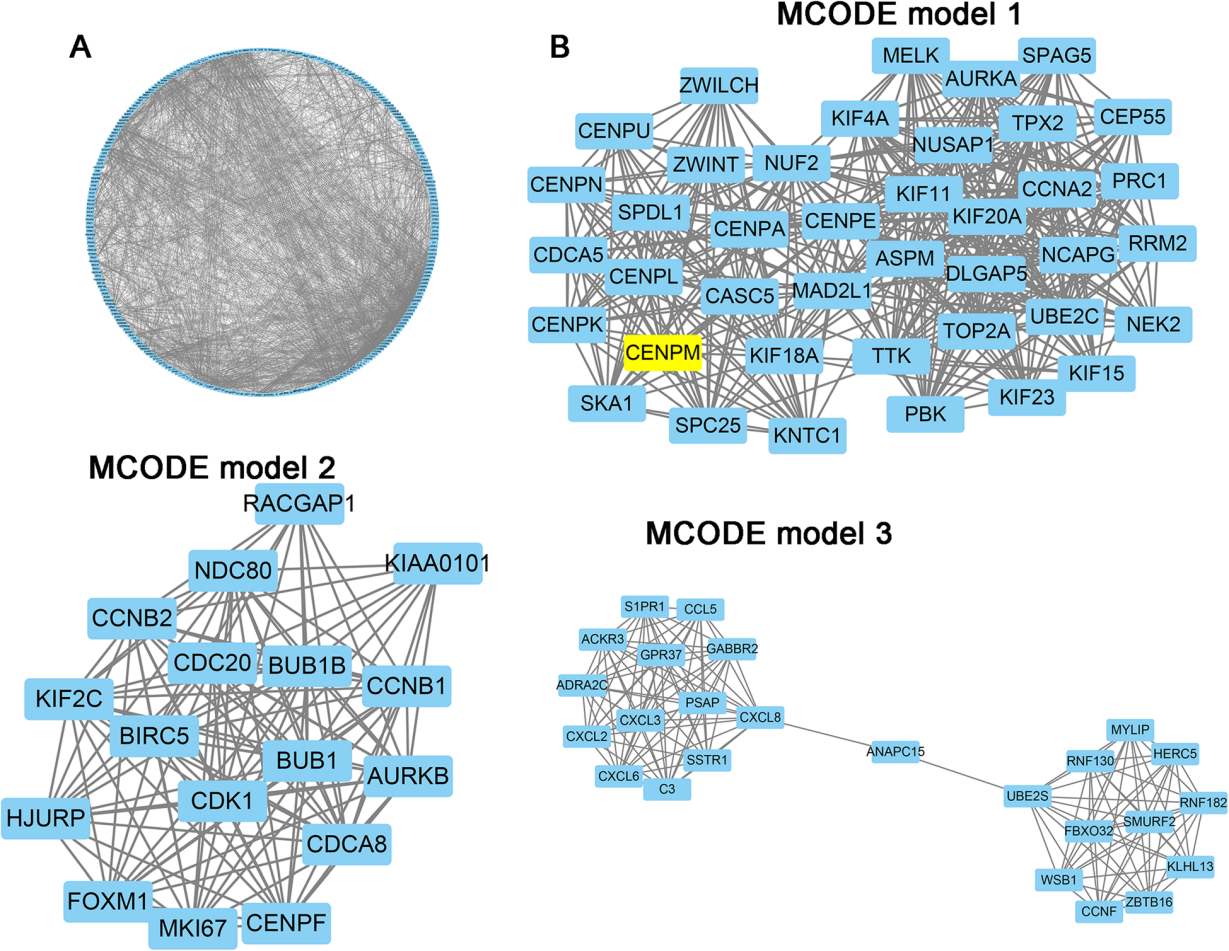
Protein-protein interaction of the differentially expressed genes. **A** Protein-protein interaction of the differentially expressed genes. **B** Module 1, MCODE score=9.238; module 2, MCODE score=8.526; and module 3, MCODE score=7.524

### Protein-protein interaction of the differentially expressed genes

Figure 4 A presents the protein-protein interaction network, which included 359 nodes and 1358 edges. Further, three functional subnet modules (MCODE model 1, MCODE model 2, and MCODE model 3) were selected from the PPI network (Fig. 4B).

### Cornuside I promoted osteogenic differentiation of BMSCs

To identify the role of Cornuside I in promoting osteogenic differentiation of BMSCs. To evaluate the effects of Cornuside I on the osteogenic responses of BMSCs in vitro, ALP and ARS were performed.

**Fig. 5:**
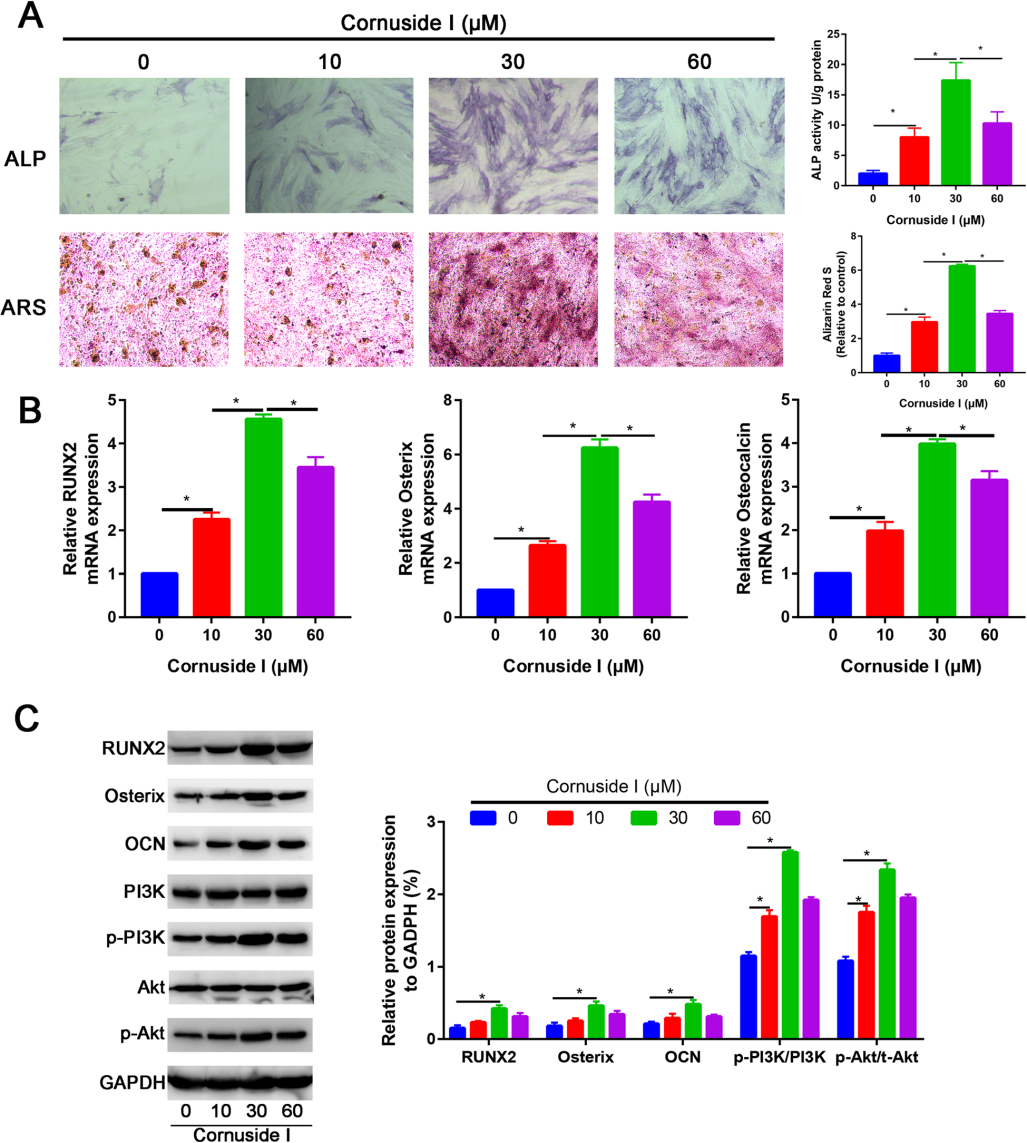
Cornuside I significantly enhanced osteogenic differentiation of BMSCs. **A** ALP staining and ARS staining of the control and Cornuside I-treated BMSCs. **B** Relative RUNX2, OSX, and Osterix mRNA expression in control and Cornuside I-treated BMSCs. **C** Relative RUNX2, OSX, and Osterix protein expression in control and Cornuside I-treated BMSCs. **D** Relative PI3K, Akt, p-PI3K, and p-Akt protein expression in control and Cornuside I-treated BMSCs

ALP and ARS results also showed that the Cornuside I (30 mM) group had higher ALP activity and calcium deposition than the control group and other dose of Cornuside I groups (Fig. 5A). Gene expression of osteogenic differentiation markers RUNX2, OSX, and CON was detected by qRT-PCR and western blot assays. Osteogenic differentiation markers RUNX2, OSX, and CON were significantly increased in Cornuside I (30 mM) group than other groups (P<0.05, Fig. 5B). Western blot analysis was in agreement with the quantitative real-time PCR (qRT-PCR) results, showing that the protein expression of RUNX2, OSX, and CON was upregulated in the Cornuside I (30 mM) group (Fig. 5C).

### Cornuside I activated the PI3K/AKT signaling pathway during osteogenic differentiation of BMSCs

To identify the mechanism of Cornuside I in promoting osteogenic differentiation of BMSCs, we firstly revealed the PI3K and Akt gene expressions in control and Cornuside I-treated BMSC groups. We found that PI3K and Akt expression were significantly upregulated in Cornuside I-treated BMSC group (P<0.05). Western blot analysis was in agreement with the quantitative real-time PCR (qRT-PCR) results, showing that the protein expression of p-PI3K and p-Akt was significantly upregulated in the Cornuside I (30 mM) group (Fig. 5C).

### LY294002 partially blocked the promotion effects of Cornuside I on osteogenic differentiation of BMSCs

**Fig. 6:**
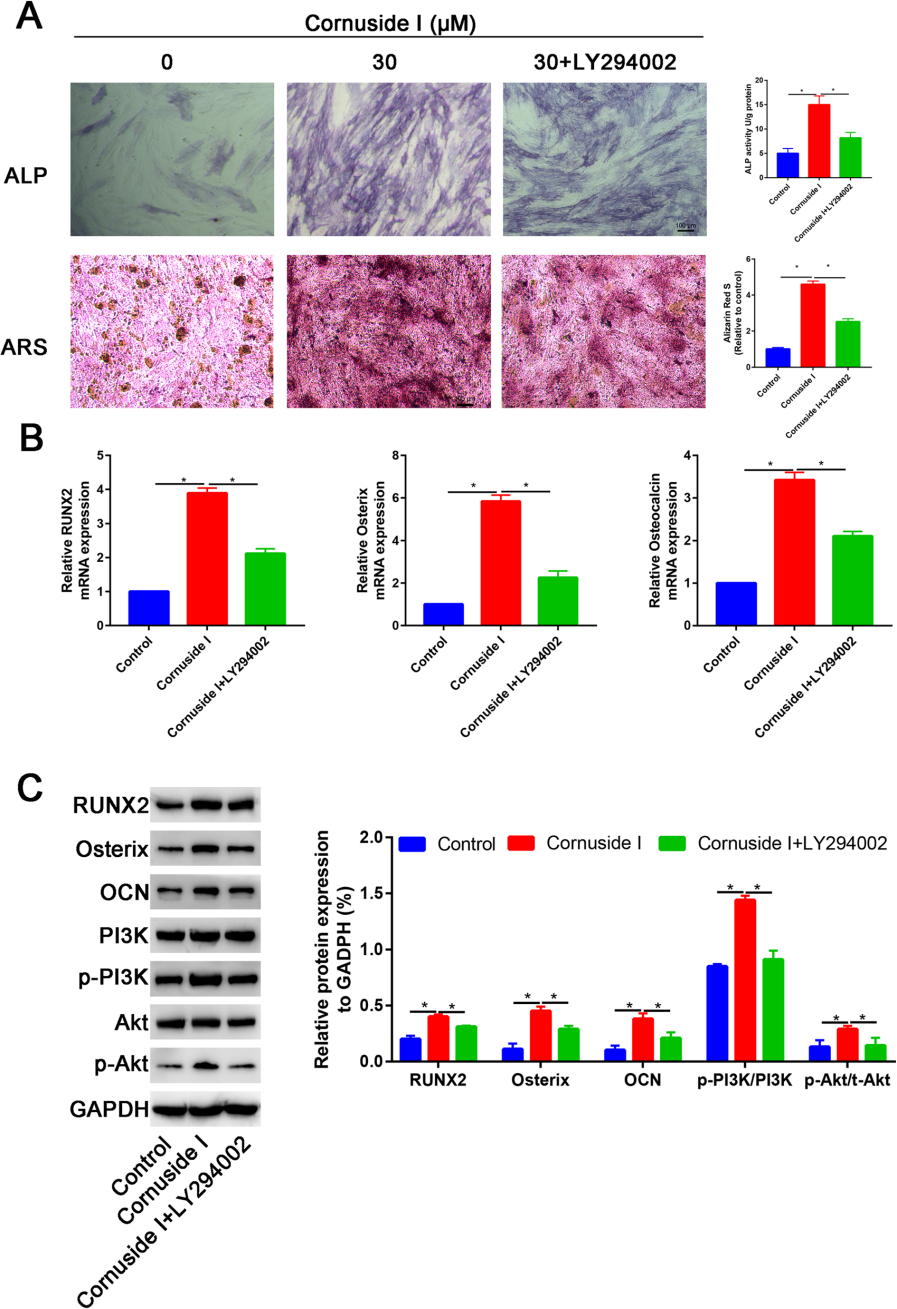
LY294002 partially blocked the promotion effects of Cornuside I on osteogenic differentiation of BMSCs. **A** ALP staining and ARS staining in control, Cornuside I, and Cornuside I + LY294002 groups. **B** Relative RUNX2, OSX, and Osterix mRNA expression in control, Cornuside I, and Cornuside I + LY294002 groups. **C** Relative RUNX2, OSX, and Osterix protein expression in control, Cornuside I, and Cornuside I + LY294002 groups. **D** Relative PI3K, Akt, p-PI3K, and p-Akt protein expression in control, Cornuside I, and Cornuside I + LY294002 groups

ALP and ARS results also showed that the Cornuside I (30 mM) group had higher ALP activity and calcium deposition than the control group and other dose of Cornuside I groups (Fig. 6A). However, the promotion effects of Cornuside I on osteogenic differentiation of BMSCs were partially blocked by LY294002 (Fig. 6A). PCR results found that Cornuside I significantly increased the PI3K and Akt expression, while LY294002 significantly downregulated the PI3K and Akt expression (Fig. 6B). Western blot analysis was in agreement with the quantitative real-time PCR (qRT-PCR) results, which suggested that LY294002 significantly downregulated the RUNX2, OSX, and CON expression (Fig. 6C).

## Discussion

This study aimed to explore the role and mechanism of Cornuside I in promoting osteogenic differentiation of BMSCs. We performed RNA sequencing to compare gene expression patterns between Cornuside I and control BMSCs. Bioinformatic analysis revealed that Cornuside I mainly affects the PI3K/Akt signaling pathway. We finally summarized that Cornuside I promotes the osteogenic differentiation of BMSCs via activation of PI3K/AKT signaling pathway.

*V. officinalis* is a traditional Chinese Medicine for nourishing the liver and kidney. Previous studies have identified that *V. officinalis* could affect the function of osteoblasts and osteoclasts and finally increase the bone mineral density. *Cornus officinalis* possess potential anti-allergic, anti-inflammatory, and antioxidant activities [20]. PI3K/AKT signaling pathway is crucial in cell proliferation, differentiation, and adaptation [21, 22]. Previous study found that PTEN/PI3K/Akt/HIF-1α pathway significantly enhanced bone regeneration in critical size defects [23]. Previous study also suggest that PI3K-AKT-mTOR signal pathway is an important regulator of the osteogenic/dentinogenic differentiation of stem cells [24].

Therefore, PI3K/AKT signaling pathway is crucial for bone formation and bone regeneration. In this study, we firstly performed RNA sequencing to identify the differentially expressed genes in Cornuside I-treated BMSCs. A total of 704 differentially expressed genes were identified between Cornuside I and normal BMSCs. These differentially expressed genes are mainly enriched in cell division and mainly participated into the PI3K/Akt signaling pathway. Therefore, we further performed ALP and ARS staining to identify the role of Cornuside I in promoting osteogenic differentiation of BMSCs. Many functions of the PI3K/Akt signaling pathway are mainly accomplished by p-Akt phosphorylating. We measured the p-PI3K and p-Akt expression in control and Cornuside I-treated BMSCs. Cornuside I significantly increased the p-PI3K and p-Akt expression than control group, which suggested that Cornuside I activated the PI3K/Akt signaling pathway. To further identify the mechanism of Cornuside I in promoting osteogenic differentiation of BMSCs. We administrated PI3K/Akt pathway inhibitor, LY294002 to further illustrate the Cornuside I on osteogenic differentiation of BMSCs. The promotion effects of Cornuside I could be partially blocked by LY294002. These results suggested that Cornuside I significantly increased the osteogenic differentiation of BMSCs through PI3K/Akt signaling pathway.

## Conclusion

In summary, this is the first study exploring the role and mechanism of Cornuside I in promoting osteogenic differentiation of BMSCs using RNA sequencing. We finally found that Cornuside I promotes the osteogenic differentiation of BMSCs via activation of PI3K/AKT signaling pathway.

## Data Availability

All the data pertaining to the present study will be willingly shared upon reasonable request.

## Abbreviations

BMSCs: Bone mesenchymal stem cells
CCK-8: Cell Counting Kit-8
ALP: Alkaline phosphatase
ARS: Alizarin Red Staining
OP: Osteoporosis
MSCs: Mesenchymal stem cells
RNA-Seq: RNA sequencing
FBS: Fetal bovine serum
DEGs: Differentially expressed genes
GO: Gene ontology
KEGG: Kyoto Encyclopedia of Genes and Genomes
BP: Biological process
MF: Molecular function
CC: Cellular component
MCODE: Molecular Complex Detection
PBS: Phosphate-buffered saline
RT-PCR: Real-time polymerase chain reaction
